# Comparative Distribution of Repetitive Sequences in the Karyotypes of *Xenopus tropicalis* and *Xenopus laevis* (Anura, Pipidae)

**DOI:** 10.3390/genes12050617

**Published:** 2021-04-21

**Authors:** Álvaro S. Roco, Thomas Liehr, Adrián Ruiz-García, Kateryna Guzmán, Mónica Bullejos

**Affiliations:** 1 Department of Experimental Biology, Faculty of Experimental Sciences, University of Jaén, Campus Las Lagunillas S/N, 23071 Jaén, Spain; asroco@ujaen.es (Á.S.R.); arg00027@red.ujaen.es (A.R.-G.); kguzman@ujaen.es (K.G.); 2 Institute of Human Genetics, Jena University Hospital, Friedrich Schiller University, Am Klinikum 1, D-07747 Jena, Germany; Thomas.Liehr@med.uni-jena.de

**Keywords:** *Xenopus tropicalis*, *Xenopus laevis*, repetitive DNA, genomic in situ hybridization (GISH), fluorescence in situ hybridization (FISH), Cot DNA, chromosome painting

## Abstract

*Xenopus laevis* and its diploid relative, *Xenopus tropicalis*, are the most used amphibian models. Their genomes have been sequenced, and they are emerging as model organisms for research into disease mechanisms. Despite the growing knowledge on their genomes based on data obtained from massive genome sequencing, basic research on repetitive sequences in these species is lacking. This study conducted a comparative analysis of repetitive sequences in *X. laevis* and *X. tropicalis*. Genomic in situ hybridization (GISH) and fluorescence in situ hybridization (FISH) with Cot DNA of both species revealed a conserved enrichment of repetitive sequences at the ends of the chromosomes in these *Xenopus* species. The repeated sequences located on the short arm of chromosome 3 from *X. tropicalis* were not related to the sequences on the short arm of chromosomes 3L and 3S from *X. laevis*, although these chromosomes were homoeologous, indicating that these regions evolved independently in these species. Furthermore, all the other repetitive sequences in *X. tropicalis* and *X. laevis* may be species-specific, as they were not revealed in cross-species hybridizations. Painting experiments in *X. laevis* with chromosome 7 from *X. tropicalis* revealed shared sequences with the short arm of chromosome 3L. These regions could be related by the presence of the nucleolus organizer region (NOR) in both chromosomes, although the region revealed by chromosome painting in the short arm of chromosome 3L in *X. laevis* did not correspond to 18S + 28S rDNA sequences, as they did not colocalize. The identification of these repeated sequences is of interest as they provide an explanation to some problems already described in the genome assemblies of these species. Furthermore, the distribution of repetitive DNA in the genomes of *X. laevis* and *X. tropicalis* might be a valuable marker to assist us in understanding the genome evolution in a group characterized by numerous polyploidization events coupled with hybridizations.

## 1. Introduction

Amphibian genomes show the greatest size variability among vertebrates, generally due to their high content of repetitive DNA and transposons [1,2,3], and due to polyploidization events [4]. The way in which changes in ploidy can affect repetitive DNA is of particular interest since polyploidization can trigger transposon activity and cause genome expansion and instability [5]. The effects on karyotype morphology and evolution are also unknown, as alterations in repetitive DNA have not been studied in depth. Amphibian chromosomes are generally large and are characterized by the absence of informative banding patterns [6,7,8,9]. This includes the sex chromosomes, which are homomorphic in most amphibian species [10,11]. In this sense, repetitive DNA can be used as chromosomal markers for comparative cytogenetic analysis [12,13] or for the identification of the sex chromosomes, as the accumulation of repetitive sequences is one of the first signs of sex chromosome differentiation according to the canonical pathway on the evolution of the sex chromosomes [14].

In situ hybridization on metaphase chromosomes using labeled genomic DNA as a probe (a technique called genomic in situ hybridization, or GISH) allows for the identification of repeated DNA sequences, as they reanneal more rapidly than unique sequences in the genome [15]. GISH can provide distinctive information about similarities between DNA from related species, as it can reveal the physical distribution of common and different sequences between the species being probed and the species used to supply the DNA probe. This method’s efficacy is largely based on genome-specific dispersed repetitive sequences (reviewed in [16]). Furthermore, if repeated DNA sequences are differentially located on sex chromosomes, this technique will allow for their identification in species with homomorphic sex chromosomes. This has been the case in some species of Lepidoptera or in the crustacean *Asellus aquaticus*, where GISH revealed the accumulation of repetitive DNA sequences on the sex chromosomes [17,18,19].

Species of the genus *Xenopus* are interesting models for studying genome evolution after polyploidization, because most changes in genome size in this genus involve changes in ploidy (ranging from diploid to dodecaploid). They are also good models to study sex chromosome evolution, as sex chromosomes have evolved independently several times in this group [20,21,22,23]. Extant species of the genus *Xenopus* are restricted to Africa and can be classified into two clades [24]. One clade includes *X. tropicalis* and all the polyploid *Xenopus* species with a number of chromosomes that are multiples of 20. The other clade is comprised of polyploid *Xenopus* species with chromosome numbers that are multiples of 18, including *X. laevis* [9]. The chromosome nomenclature initially proposed for *Xenopus* species (including *X. tropicalis* and *X. laevis*) was based on relative sizes of p and q arms [25,26] (for a review on *Xenopus* cytogenetics, see [27]). Although this nomenclature has been widely used [6,28,29], a change based on chromosome size was first established by [30], promoting the renumbering of the genetic linkage groups in the v4 draft of the *X. tropicalis* genome assembly [31]. The currently accepted nomenclature of *Xenopus* chromosomes was established by the *Xenopus* Gene Nomenclature Committee and is based on chromosome size and phylogenetic relationships [9,32].

Among *Xenopus* species, *X. tropicalis* is an interesting model as it is the only diploid species of the genus, with a karyotype containing 2n = 2x = 20 chromosomes [25]. Furthermore, in this species, three homomorphic sex chromosomes coexist (Y > W > Z) both in laboratory strains and in natural populations [20,21]. In contrast, *X. laevis* is a functional diploid with an allotetraploid origin. Accordingly, two subgenomes, L and S, can be identified based on the size of the homoeologous chromosomes (long and short) and on the differential accumulation of transposon families in each one (2n = 4x = 36 chromosomes) [33,34]. About 25–30% of the genome in *X. laevis* is comprised of sequences repeated more than 100 times [33], although no evident sex-specific accumulation of repetitive sequences has been identified on the homomorphic sex-chromosomes (ZZ/ZW) of this species [6,28].

According to genetic mapping, *X. laevis* and *X. tropicalis* chromosomes have maintained conserved synteny since their divergence around 48 Mya [28,33,35]. The major chromosome rearrangement observed is the fusion of chromosomes 9 and 10 (about 48–34 Mya) in the ancestor of the two extinct progenitor species that led to *X. laevis* by hybridization, followed by polyploidization, about 17–18 Mya [6,33]. Other mayor chromosome rearrangements (translocations, insertions, deletions, inversions or sex-specific replication bands) have not been identified in these species [9], although inversions are observed in subgenomes L and S from *X. laevis* [33].

The sex chromosomes in these species are homomorphic, with no heteromorphism identified so far [20,28,36,37]. Furthermore, the sex chromosomes in *X. laevis* and *X. tropicalis* are not homoeologous. The sex-determining locus in *X. laevis* (*dm-w* [38]) is located at the end of the long arm of chromosome 2L (XLA2L), while the sex-determining locus in *X. tropicalis* is located at the end of the short arm of chromosome 7 (XTR7). XTR7 is also of interest, as silver staining in metaphase chromosomes identified a single active nucleolus organizer region (NOR) located in the secondary constriction of 7q [25,39]. In some amphibian species, pairs carrying NORs have been linked to sex chromosomes (*Gastrotheca riobambae* [40], *Leiopelma hamiltoni* [41], *Hyla femoralis* [42], *Buergeria buergeri* [43], *Bufo marinus* [44]). In this sense, the comparative study of sequences of this chromosome (XTR7) on other *Xenopus* species is of interest for the study of the evolution of NOR-bearing chromosomes and sex chromosomes in the genus *Xenopus*.

In this study, we used GISH and FISH with Cot DNA to compare the distribution of repetitive sequences in the species *X. tropicalis* and *X. laevis*, diploid and allotetraploid representatives of the genus *Xenopus*. Furthermore, chromosome 7 from *X. tropicalis* was applied for chromosome painting experiments to establish common sequences between this NOR-bearing sex chromosome and *X. laevis* chromosomes. The study of common and specific repetitive sequences in species of this genus will provide information about the evolution of genomes and chromosomes after hybridization and polyploidization events.

## 2. Materials and Methods

### 2.1. Animals

*X. tropicalis* and *X. laevis* were purchased from Xenopus Express (Rennes, France) and maintained at the Centro Andaluz de Biología del Desarrollo (CABD) (Seville, Spain). Tadpoles from these species were a generous gift from Jose Luis Gómez-Skarmeta. All animal protocols were approved by the ethics committee for research on animals of the University of Jaén and authorized by the competent authority (project number 30-11-15-375). The care and treatment of animals used in this research was conducted in accordance with policies on animal care provided by Spanish and EU regulations.

### 2.2. Cell Culture, Chromosome Preparations and Banding Analyses

Primary cell cultures were prepared from tadpole limbs as described in [45]. Briefly, stage 55 tadpoles (according to Nieuwkoop and Faber [46]) were euthanized by immersion in 2 g/L bicarbonate-buffered tricaine methanesulfonate (MS-222) (Sigma Aldrich, Darmstadt, Germany) in water, washed in 70% ethanol, and their gonads and limbs were dissected in PBS. Gonads were fixed in Bouin’s solution, embedded in paraffin and processed to establish their sex by histological analysis. Limbs were washed in clean PBS (Sigma Aldrich, Germany), transferred into sterile tubes, disaggregated with sterile scissors and then cultured in DMEM (Sigma Aldrich, Germany) supplemented with 10% fetal calf serum (PAA Laboratories, Cölbe, Germany), 100 µg/mL penicillin, 100 U/mL streptomycin and 2.5 µg/mL amphotericin B (all antibiotics from Sigma Aldrich, Germany). Cell cultures were maintained in 25 cm^2^ tissue culture flasks at 28 °C under ordinary atmospheric conditions. Confluent primary and secondary cultures were subsequently transferred to 75 cm^2^ tissue culture flasks.

Mitotic chromosomes from *X. tropicalis* (three females and two males) and *X. laevis* (three females) were obtained from secondary cultures as described previously [47]. Briefly, colcemid (Invitrogen, Waltham, MA, USA) was added to the culture medium at a final concentration of 0.1 µg/mL. After 5 h of incubation, cells were harvested, centrifuged and treated with a hypotonic solution (0.4% KCl) for 20 min. The cell suspension was fixed in methanol and acetic acid (3:1 *v*/*v*), washed three times with new fixative and dropped onto microscopic slides. 

Triple staining chromomycin A_3_ (CMA_3_)/distamycin A (DA)/4′, 6-diamidino-2-phenylindole dihydrochloride (DAPI) (Sigma Aldrich, Germany) was performed according to [48]. Briefly, the slides were stained with CMA_3_ (0.5 mg/mL in McIlvaine buffer, pH 7.0, containing 10 nM MgCl_2_) for 60 min, washed with distilled water, stained with DA (0.1 mg/mL) for 30 min, washed again and stained with DAPI (0.5 mg/mL) for 30 min. Finally, the slides were washed with distilled water, air dried and mounted with Vectashield anti-fade medium (Vector Laboratories, USA). Samples were examined under a fluorescent microscope. Nucleolus organizing regions (NOR) were detected by AgNO_3_ staining according to [49]. For the chromosome nomenclature of *X. tropicalis* and *X. laevis* used in this work, see [9,32].

### 2.3. Chromosome Microdissection

Chromosome microdissection was carried out in an inverted microscope (Zeiss Axiovert 200) using glass needles attached to an electronic micromanipulator. Glass needles were made from 2 mm diameter glass capillaries using a vertical pipette puller (Narishige PB-7). Chromosome preparations used for microdissection were obtained from a cell culture derived from a female tadpole (the probable sex chromosome constitution is ZW, although WW cannot be ruled out). Fresh, dry chromosome extensions were prepared on 24 × 60 mm coverslips, previously washed with 10% SDS and distilled water. About 15–20 microdissected chromosomes were transferred to a glass micropipette containing the collection solution (10 mM Tris-HCl pH 7.5, 10 mM NaCl, 0.1% SDS, 1 mM EDTA pH 7.5–8.0, 0.1% Triton X-100, 1.44 mg/mL proteinase K (Applichem, Darmstadt, Germany) and 30% glycerol) and then incubated for 1 h in a wet chamber. For the technical details of glass needle-based microdissection, see [50].

### 2.4. Probe Synthesis and Labelling

Genomic DNA was labeled with biotin 11–dUTP (Sigma Aldrich, Germany) using a nick-translation kit (Sigma Aldrich, Germany). Three probes were obtained, depending on the species and sex of the DNA sample used (*X. tropicalis* ZZ male, *X. tropicalis* WW female and *X. laevis* ZZ male). The length of the probe was between 150 and 1000 bp. Cot-1 DNA from *X. laevis* (ZZ male) and Cot-1 to Cot-20 from *X. tropicalis* (mix of ZW females) were prepared according to [51] and labeled with biotin 11–dUTP using nick-translation in the same way as genomic DNA, or with fluorochromes (SpectrumGreen–dUTP or Texas Red–dUTP (Vysis, Richmond, UK)) using the DOP-PCR method [52]. The telomere probe was synthetized and labeled with biotin–dUTP as part of the same PCR reaction using the oligos Telo1 (TTAGGG)_5_ and Telo2 (CCCTAA)_5_ and the following thermal program: 94 °C × 5 min; (94 °C × 1 min; 55 °C × 30 s; 72 °C × 30 s) × 10 cycles; (94 °C × 1 min; 60 °C × 30 s; 72 °C × 30 s) × 35 cycles; 72 °C × 5 min. The plasmid pDmra51#1, containing rDNA from *Drosophila melanogaster* [53], was labeled by DOP-PCR with Texas Red–dUTP and used as a probe for the direct detection of ribosomal DNA.

Chromosome painting probes were obtained from needle-microdissected chromosomes 7 and 7p from *X. tropicalis*. After proteinase K treatment, microdissected chromosomes were pre-amplified using sequenase (USB, Cleveland, OH, USA) in 0.63 μL of sequenase buffer, 0.4 μL of 0.2 mM dNTPs, 0.6 μL of 40 mM DOP primer (5′-CCGACTCGAGNNNNNNATGTGG-3′) and 3.37 μL of PCR water per sample. Pre-amplification by DOP-PCR was conducted using the following program: 92 °C for 5 min (to inactivate proteinase K), followed by eight cycles of 90 °C for 1 min, 25 °C for 2 min and 34 °C for 2 min. Due to enzyme inactivation during the denaturation step, 0.2 μL of sequenase mix (12 U/μL of sequenase and 1.75 μL of sequenase dilution buffer) was added in each cycle during the annealing step. The first round of amplification was performed by adding 0.1 U Taq polymerase (Bioline GmbH, Luckenwalde, Germany), 0.2 mM dNTPs, 20 μM DOP primer, 25 mM MgCl_2_ and 34.23 μL of PCR water (final volume of 50 μL) under the following conditions: 33 cycles of 92 °C for 1 min, 56 °C for 2 min, 72 °C for 2 min, with the addition of a final extension step of 5 min at 72 °C. In the second round of 30-cycle DOP-PCR, painting probes were labeled with Texas Red–dUTP (Vysis, Richmond, UK) using 1 μL of the previous DOP-PCR products as template DNA. Painting probes were named XTR-7w (probe from the whole of chromosome 7 from *X. tropicalis*) and XTR-7p (probe from the short arm of the same chromosome) [50].

### 2.5. Fluorescence In Situ Hybridization (FISH) and Chromosome Painting

Chromosome preparations were incubated in RNaseA (Roche, Mannheim, Germany) solution (100 µg/mL in 2xSSC) for 1 h at 37 °C, washed three times in 2xSSC for 5 min, and then incubated in Pepsin (Applichem) solution (50 µg/mL in 0.01 N HCl) at 37 °C for 5 min. After two washes in 2xSSC for 5 min, the slides were fixed in 1% formaldehyde (Applichem) (*v*/*v*) in PBS for 10 min, washed 3 times for 5 min in 2xSCC, dehydrated via an ethanol series (70, 90 and 100%, 5 min each) and air dried.

Chromosomes were denatured at 70 °C for 3 min in 70% formamide/2xSSC, washed in 2xSSC, dehydrated via an ethanol series and air dried. For each slide, the hybridization solution (containing 200 ng of labeled DNA dissolved in 10 μL of 50% formamide/2xSSC; 15 μg of Cot-1 DNA) was denatured at 73 °C for 6 min and then cooled on ice (chromosome painting probes were left at 37 °C for 30 min for renaturalization before cooling on in ice). For hybridization, 10 μL of the denatured probe was added to the slide and covered with a coverslip. Slides were left overnight in a moist chamber at 37 °C.

Post-hybridization washes for direct fluorescence included one wash with 2xSSC for 5 min at room temperature; one wash of 2 min in 0.3% Nonidet P40 (Roche, Mannheim, Germany), 0.4xSSC at 65 °C and one wash of 30s in 0.1% Nonidet P40/2xSSC at room temperature. Finally, slides were dehydrated in 90% and 100% absolute ethanol for 5 min each, dried and mounted in anti-fade solution (Vectashield with DAPI) (Vector Laboratories, Burlingame, CA, USA).

Post-hybridization washes for indirect fluorescence included one wash of 5 min in 2xSSC at room temperature; three washes of 5 min each in 50% formamide/2xSSC at 37 °C; two washes of 5 min each in 2xSSC at room temperature and one wash of 5 min in 4T (4xSSC, 0.05% Tween 20). Slides were blocked for 1 h in 4M (5% blocking reagent (Roche) in 4T) in a humid chamber at room temperature, washed for 5 min in 4T, incubated in fluorescein isothiocyanate (FITC)-conjugated avidin (Vector Laboratories, Burlingame, CA, USA) (1:1000 in 4T) and then washed three times (5 min each) in 4T. For signal amplification, slides were incubated in biotinylated avidin (Vector Laboratories, USA) (1:100 in 4M) for 20 min, washed three times (10 min each) in 4T, followed by a second incubation in avidin—FITC (1:1000 in 4T) for 20 min and three washes (10 min each) in 4T. Finally, slides were washed four times (5 min each) in PBS, dehydrated via an ethanol series, air dried and mounted in anti-fade solution (Vectashield with DAPI) (Vector Laboratories, Burlingame, CA, USA).

### 2.6. Microscopy and Image Capture

About 10–25 metaphases were analyzed in each experiment (NOR, triple staining, GISH, FISH with Cot DNA and chromosome painting). Chromosome images were captured with a digital CCD camera (Olympus DP70) coupled with a fluorescence microscope (Olympus BX51). Images were processed further with Adobe Photoshop CS2 software.

## 3. Results and Discussion

### 3.1. Genomic In Situ Hybridization (GISH)

**Figure 1:**
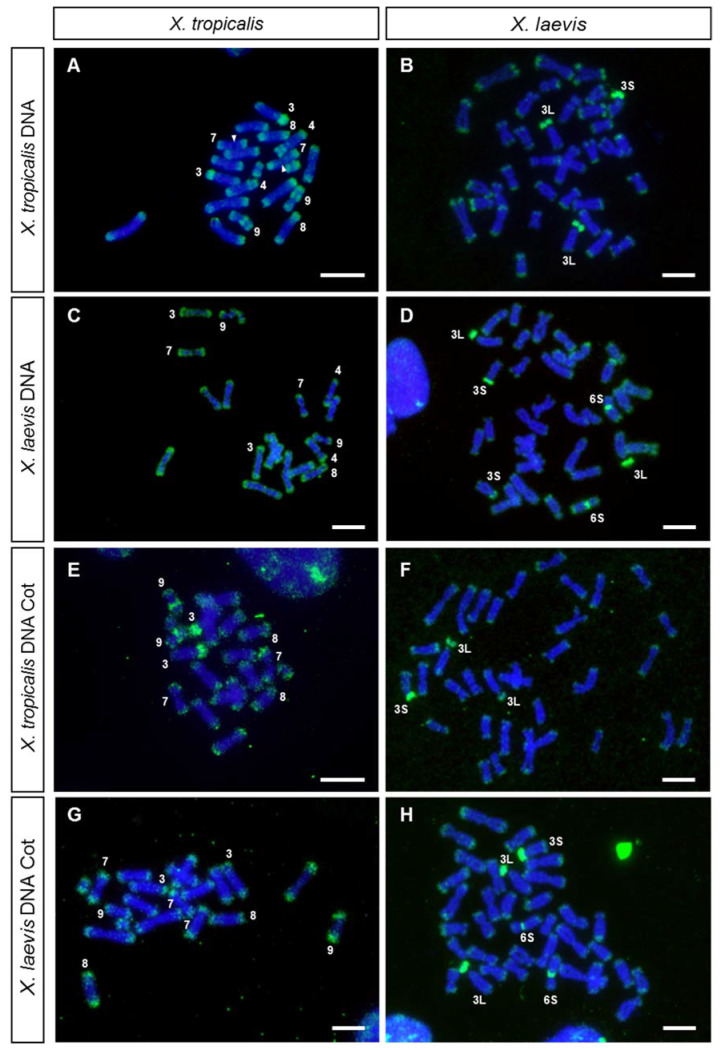
GISH and FISH with Cot DNA. Metaphase chromosomes from *X. tropicalis* (**A**,**C**,**E**,**G**) and *X. laevis* (**B**,**D**,**F**,**H**) hybridized using, as a probe: (**A**,**B**) genomic DNA from *X. tropicalis* (female), (**C**,**D**) genomic DNA from *X. laevis* (male), (**E**,**F**) Cot DNA from *X. tropicalis* (female) and (**G**,**H**) Cot DNA from *X. laevis* (male). All metaphases were derived from female individuals. All probes were labeled with biotin 11–dUTP, and three rounds of amplification were used during immunological detection. The arrowheads point to the NOR. Chromosome XTR8 is mounted in C. Scale: 5 µm.

**Figure 2:**
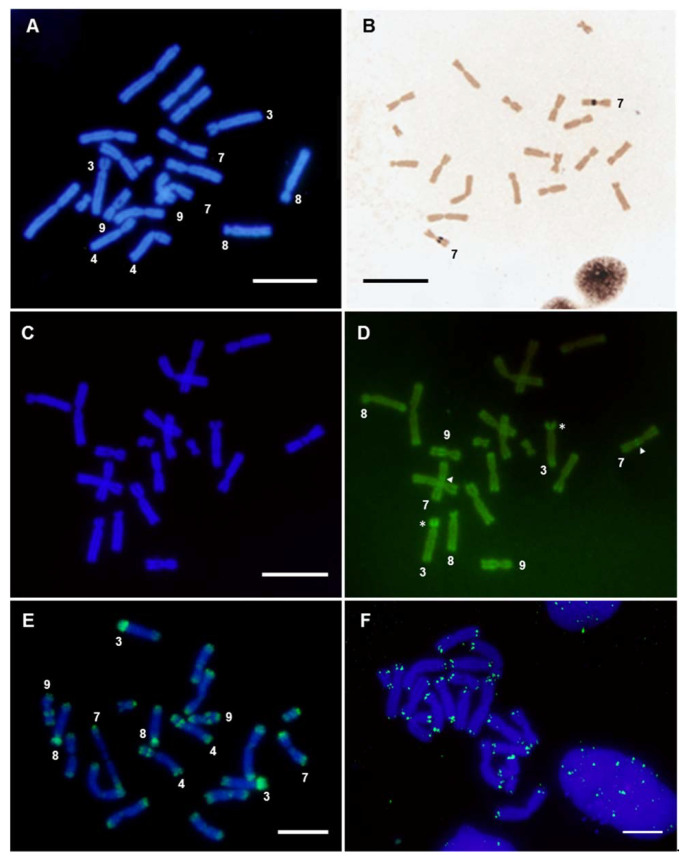
Analysis of *X. tropicalis* chromosomes in female samples. (**A**) DAPI-stained metaphase from *X. tropicalis*. (**B**) Ag-NOR staining revealing active NOR located on the secondary constriction of chromosome 7. (**C**,**D**) Triple staining using CMA_3_/DA/DAPI. (**C**) Metaphase spread from *X. tropicalis* using DAPI filter; (**D**) the same metaphase using a FITC filter. The arrow points to the secondary constrictions on pair 7 (NOR). The asterisk (*) signals the short arm of XTR3. (**E**) In situ hybridization on *X. tropicalis* metaphase chromosomes using *X. tropicalis* SpectrumGreen-labeled Cot-20 DNA as a probe (direct detection). (**F**) In situ hybridization using a telomeric probe, labeled with biotin 11–dUTP (after three rounds of amplification). Scale: 5 µm.

The hybridization of metaphase chromosomes from *X. tropicalis* with labeled genomic DNA from *X. tropicalis* showed positive signals at the ends of all chromosomes of the karyotype (Figure 1A and Figure S1). Intense signals were also observed on the short arm of chromosome 3, which stained completely, on the secondary constriction of chromosome 9 and on the C-positive band [39] of chromosome 4 (chromosome 5 according to [30]), but not on the secondary constriction of chromosome 7, where the nucleolar organizing region (NOR) is located (Figure 1A and Figure 2A,B). Most positive regions revealed by GISH were also positive when stained with CMA_3_ (a GC-specific dye [54])—chromosome ends, the 3p arm and the secondary constriction of chromosomes 7 (faint) and 9 (Figure 2C,D)—revealing that these regions are GC rich. No sex-linked difference was observed when male or female chromosomes were hybridized with male or female probes (Figure S2).

The hybridization pattern observed when genomic DNA was used as probe was similar to that obtained with labeled Cot DNA (Cot-1 to Cot-20) from *X. tropicalis* (Figure 1E, Figure 2E and Figure S3), confirming that the positive signals correspond to repetitive DNA sequences. It should be noted that, using direct fluorescence, labeled Cot DNA also identified a strong signal on the short arm of chromosome 8 and lower intensity signals at centromeric or pericentromeric positions (Figure 2E and Figure S3).

*X. laevis* metaphase spreads, hybridized with labeled genomic DNA from *X. laevis*, showed a hybridization pattern different to that observed in *X. tropicalis* (Figure 1D). In metaphase spreads from *X. laevis*, the signals at the ends of the chromosomes were less intense than those observed in *X. tropicalis*, and positive signals were detected on the short arm of chromosome 3L (except for the region of the secondary constriction, corresponding to the only NOR of the karyotype of this species), on the centromere of pair 6S and on the short arm of one acrocentric pair. This could be the pair 3S, as it has a strong C-positive band in its short arm [6,55] and it is homoeologous of chromosome 3L [6,28,29]. However, it could also be the chromosome pair 4L since, according to [6,55], it also has a strong C-positive band in its short arm. The hybridization pattern observed after GISH coincided with that observed when DNA Cot-1 from *X. laevis* was used as probe (Figure 1H). Noteworthily, differences in the size of the signals between homologous chromosomes existed on the short arm of chromosome 3S (or 4L) and on the centromere of pair 6S. These differences were observed in three female siblings (Figure S4), and could be due to polymorphisms for the size of the repetitive region. Further analysis using unrelated individuals will be necessary to confirm the existence of such polymorphisms. On the other hand, samples from female tadpoles (ZW), showed no differences in the hybridization pattern when both chromosomes 3L (sex chromosomes) were compared (Figure 1B,D,F,H).

The relationships between the repetitive DNA sequences from *X. tropicalis* and *X. laevis* were analyzed by cross-hybridization, that is, metaphase spreads from one species were hybridized with genomic DNA or Cot-1 DNA from the other species, and vice versa (Figure 1B,C,F,G). When *X. laevis* genomic DNA was probed on *X. tropicalis* chromosomes, the hybridization signals were detected at the end of all the chromosomes of the karyotype (Figure 1C), with a pattern similar to that observed when genomic DNA from *X. tropicalis* was used as a probe (Figure 1A). Nevertheless, in this case, no signals were observed on the secondary constriction of pair number 9, the short arm of chromosome 4 or the short arm of acrocentric pair 3. On the other hand, when *X. laevis* chromosomes were hybridized with genomic DNA from *X. tropicalis* (Figure 1B), the hybridization signals were detected at the ends of all chromosomes of the karyotype (but with a lower intensity than in metaphase chromosomes from *X. tropicalis*) and on the short arms of chromosomes 3L and 3S (or 4L). However, no positive centromeric signal was observed on pair number 6S. The hybridization pattern obtained by GISH was similar to that observed when *X. tropicalis* or *X. laevis* Cot-1 DNA were used as probes (Figure 1G,H).

When GISH or Cot DNA hybridization experiments were performed with probes and chromosomes from *X. tropicalis*, the ends of all chromosomes were revealed and stained with high intensity. This hybridization pattern demonstrates a rather interesting distribution of repeated DNA sequences, also present in the *X. laevis* karyotype, although amplified to a smaller extent. The intensity of the signal observed at the ends of chromosomes using labeled genomic DNA or Cot DNA as probes did not correspond exclusively to telomeric repeats (TTAGGG)n, as the signal obtained after hybridizing with telomeric sequences was smaller in size (Figure 2F). This indicates that these hybridization signals were not solely due to telomeric sequences, but also due to sub-telomeric repetitive DNA with differential accumulation in these species.

The existence of repeated DNA sequences at the ends of chromosomes has been described in *Glandirana rugosa*, a species where two types of short repeats (41 and 31 bp) have been located at the ends of chromosomes [56]. However, although searched, no similar sequences have been found in the genome of *X. tropicalis*, so they must not be related [56]. GISH experiments in other amphibian species have not revealed a similar distribution of repeated sequences at chromosome ends (*Bufo bufo*, *Epidalea calamita*, *Bufotes viridis*, and *Pelobates cultipes*), although accumulations of repeated DNA at other locations have been identified in some species (unpublished data).

The terminal/subterminal regions of *X. tropicalis* and *Pipa carvalhoi* (an anuran species of the same family) chromosomes have been found to be enriched in microsatellite motifs [12]. Furthermore, in *X. laevis*, the repetitive DNA of 77–79 bp [57] has been predominantly located in chromosome ends [9]. These characteristics have also been observed in the karyotypes of fish [58] and birds [59]. Thus, the accumulation of microsatellites and repetitive sequences in these regions may reflect their role in the organization and function of these chromosomal regions [60,61].

Regarding other chromosomal locations, GISH and FISH with Cot DNA on *X. tropicalis* chromosomes also intensely stained the short arms of the acrocentric chromosomes (XTR3 and XTR8), as well as the secondary constrictions of XTR9 and XTR4 (Figure 1A,E and Figure 2E). These regions are C-positive [39] and are very late replicating in the case of the short arm of XTR3 and the secondary constriction of the short arm of XTR4 [9]. On the other hand, GISH and FISH with Cot DNA on *X. laevis* chromosomes revealed positive signals at the short arm of pair XLA3L, the short arm of pair XLA3S (or XLA4L) and the centromeric region of pair XLA6S (Figure 1D,H). Again, this pattern of hybridization is not surprising considering the C-banded karyotype described in *X. laevis*, with C-positive bands precisely at these positions [6].

The repeated sequences on the short arm of XLA3L and XLA3S are of interest, since they were revealed with probes from both species, indicating these sequences are present in *X. tropicalis* and *X. laevis*. Nevertheless, their distribution in the karyotype of these species must be different, since probes from *X. laevis* did not reveal accumulations of repetitive sequences in the chromosomes of *X. tropicalis*, apart from the sub-telomeric repeats. Regarding the repetitive sequences located on the short arm of XTR3 (homologous to XLA3L and XLA3S), they were revealed with probes from *X. tropicalis*, but not from *X. laevis*. Thus, the repeated sequences located on the C-positive regions of chromosomes 3, 4, 8 and 9 from *X. tropicalis* and on chromosome 6S from *X. laevis* may correspond to species-specific repeated sequences. Testing the relationships between these sequences will require their isolation to use them as probes in chromosome painting experiments in several species of the genus *Xenopus*. Interestingly, painting experiments with XTR3 on *X. laevis* revealed positive signals on the long arms of XLA3L and XLA3S, but no intense signals were evident on the short arms of these chromosomes, even though the short arm of XTR3 was strongly stained with the same probe [29]. Taken together, these results reveal differences in the repetitive sequences located on the short arm of XTR3 and those present on the short arms of XLA3L and XL3S, indicating that these regions are evolving independently in these species.

The distribution of repetitive sequences revealed by GISH and FISH with Cot DNA may help in the interpretation of mapping and sequencing results. Repeated DNA sequences in these *Xenopus* species have hampered the assembly of their genomes. In fact, the genetic map used to produce the v9.0 chromosome scale assembly in *X. tropicalis* reveals low levels of recombination and a scarcity of genetic markers, specifically in the short arms of XTR3 and XTR8 [62]. Increasing our knowledge about these repeated DNA sequences and their chromosomal organization could help to improve the genome assembly of these species.

### 3.2. Chromosome Painting

**Figure 3:**
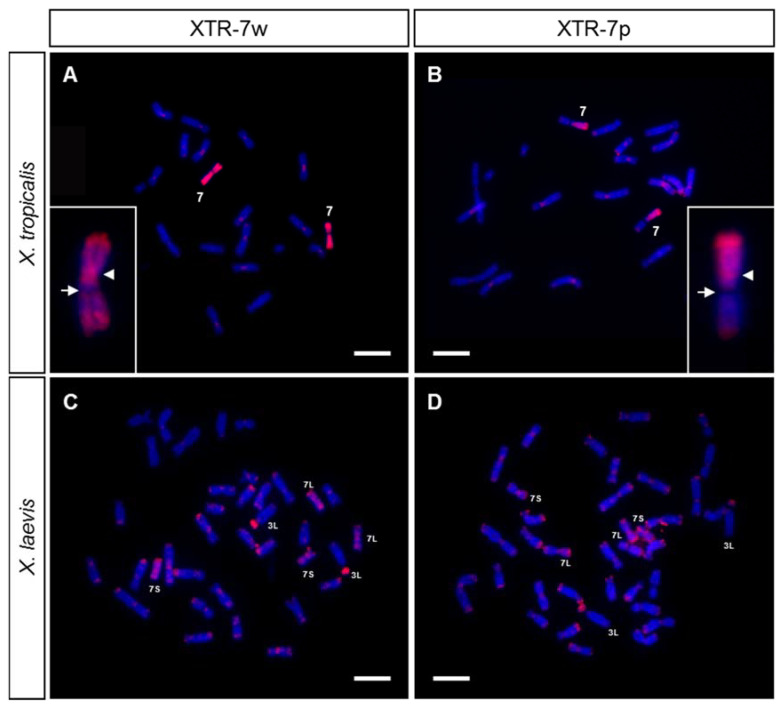
Chromosome painting with XTR-7 probes on *X. tropicalis* (**A**,**B**) and *X. laevis* (**C**,**D**) metaphase chromosomes from female samples, using two painting probes from *X. tropicalis* labeled with Texas Red–dUTP: XTR-7w (**A**,**C**) or XTR-7p (**B**,**D**). The insert in A and B shows chromosome 7 from *X. tropicalis* at a higher magnification after hybridization with XTR-7w or XTR-7p, respectively. Note the absence of hybridization signal on the secondary constriction (NOR) of chromosome 7. The arrowhead points to the centromere; the arrow points to the NOR. The signals observed are from direct fluorescence. Scale: 5 µm.

The active NOR in *X. tropicalis* was located in the secondary constriction of 7q (Figure 2B and [25,39]), while the sex determining locus was at the end of 7p [20,36,37]. Using chromosome microdissection, two painting probes from *X. tropicalis*’ chromosome 7 were obtained: the whole of chromosome 7 (XTR-7w) and the short arm of chromosome 7 (XTR-7p). These probes were labeled by DOP-PCR and used in chromosome painting experiments on metaphase chromosomes from *X. tropicalis* and *X. laevis*. When these probes (XTR-7w and XTR-7p) were hybridized with *X. tropicalis* metaphase spreads, the expected signals were observed in each case: the whole of chromosome 7 or only the short arm were painted, respectively (Figure 3A,B). The NOR was not stained when an XTR-7w probe was used, probably due to the inefficient amplification of these sequences by DOP-PCR when heterogeneous DNA samples were used as templates (rDNA was labelled by DOP-PCR using plasmid pDmra51#1 as a template). Positive signals were also observed in telomeric and some centromeric regions of most chromosomes of the karyotype. Since their intensity could be reduced by adding unlabeled Cot-1 DNA from *X. tropicalis* to the probe, these signals may be caused by repetitive telomeric and centromeric sequences. Furthermore, since XTR-7w and XTR-7p probes painted almost all telomeric regions, similar repetitive sequences must be involved in the organization of these regions.

When XTR-7w and XTR-7p probes hybridize on metaphase chromosomes from *X. laevis*, intense signals were detected on the chromosome pairs 7L and 7S, which were painted completely with the XTR-7w probe, but only on the short arm if the probe used was XTR-7p (Figure 3C,D). Both probes also revealed intense signals on the short arm of chromosome 3L, and dispersed signals in most telomeric and some centromeric regions. Centromeric and telomeric signals could be reduced by adding unlabeled Cot-1 DNA from *X. tropicalis* to the hybridization solution.

The signals obtained with the XTR-7w and XTR-7p probes in *X. laevis* agree with those previously reported by [29]. The existence of hybridization signals in two chromosome pairs (7L and 7S) from *X. laevis* with a painting probe from a single chromosome in *X. tropicalis* was expected considering the allotetraploid origin of *X. laevis* [24], and it shows that these chromosomes are homologous to XTR7.

Painting experiments also confirmed that the sex chromosomes of both species have an independent origin, confirming previous reports by [29]. Chromosome 7 is the sex pair in *X. tropicalis* [20,36,37], but not in *X. laevis*, where the homomorphic ZZ/ZW sex chromosomes correspond to pair 2L [28]. Thus, after the divergence of the two clades of *Xenopus* from a common ancestor about 20–60 million years ago, these species used different sex-determining genes located on different chromosomes. This is evident by the comparative mapping between *X. laevis* and *X. tropicalis*, as the genes located on *X. tropicalis* chromosome 7 (sex pair) were located on chromosomes 7L and 7S of *X. laevis*. Similarly, genes on the sex chromosome pair of *X. laevis* (2L) were related to those of chromosome 2S in *X. laevis* (due to their allotetraploid origin) and to chromosome 2 in *X. tropicalis* [35].

**Figure 4:**
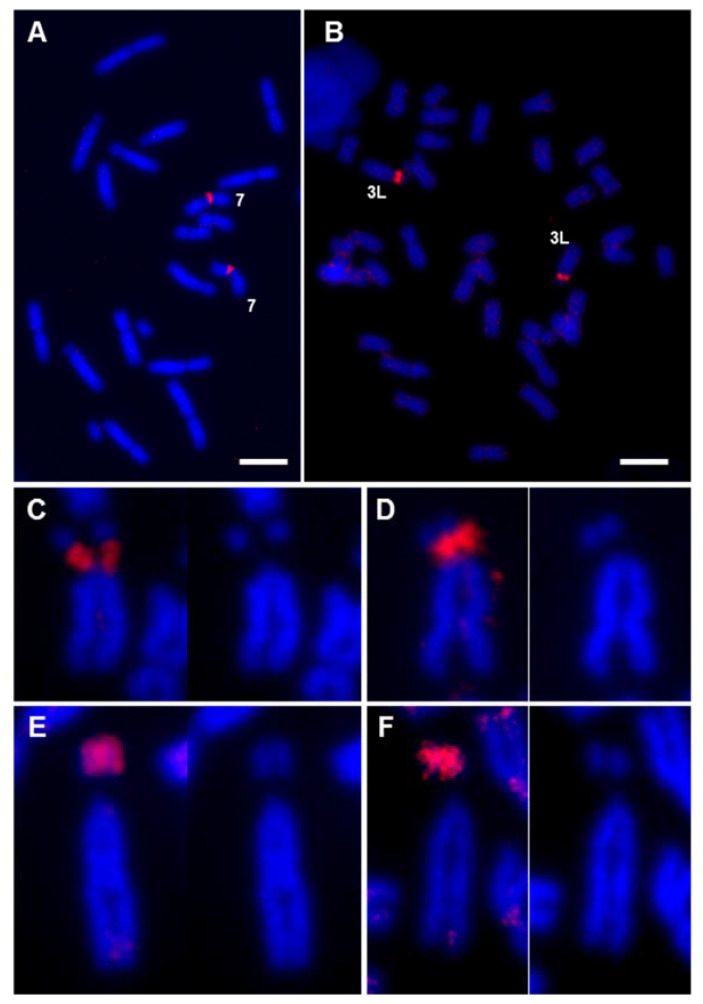
FISH with rDNA probe vs chromosome painting with XTR-7. Metaphase chromosomes from female *X. tropicalis* (**A**) and male *X. laevis* (**B**) samples, hybridized with an rDNA probe (18S + 28S) labeled with Texas Red–dUTP. (**C**–**F**) Detail of chromosome 3L from *X. laevis* hybridized with the rDNA probe (**C**,**D**) and with the painting probe XTR-7w (**E**,**F**). In all cases, the stained chromosome was compared with the image of the same chromosome stained only with DAPI. The image comparison revealed that the hybridization signal with the ribosomal DNA probe, which was located in the region of the secondary constriction, did not coincide with the hybridization signal when XTR-7w was used as a probe, which was located at the end of the short arm of the chromosome. All signals correspond to direct fluorescence. Complete metaphases corresponding to Figure 4C–F are depicted in Figure S5. Scale: 5 µm.

The intense painting signal observed on the short arm of chromosome 3L from *X. laevis* could be explained by the presence of rDNA sequences [29], since chromosome 7 from *X. tropicalis* and chromosome 3L from *X. laevis* are the only chromosomes of the karyotype of these species where the NOR is located [6]. However, the XTR-7w painting probe did not paint the NOR in *X. tropicalis*. To rule out the possibility that the XTR-7w probe was able to paint the NOR in *X. laevis*, a probe containing 18S and 28S ribosomal DNA sequences from *D. melanogaster* was hybridized with *X. tropicalis* and *X. laevis* chromosomes (Figure 4A,B, respectively). The hybridization signal in *X. tropicalis* and *X. laevis* was located on the secondary constriction of pairs 7 and 3L, respectively (Figure 4A,B). A close comparative examination of chromosome 3L in *X. laevis* revealed differences between the hybridization signals obtained with the rDNA probe (Figure 4C,D) and the XTR-7w painting probe (Figure 4E,F). According to these results, the evidence indicates that the signal detected on the short arm of XLA3L after chromosome painting with XTR-7w was not due to rDNA sequences.

Firstly, when XTR-7w was used as a probe on metaphase chromosomes of the same species, no hybridization signal was observed on the secondary constriction of XTR7 (the position where the rDNA is located, according to FISH experiments). This indicates that the probe was not enriched on rDNA sequences, perhaps due to the inefficient amplification of these sequences by DOP-PCR when a complex mixture was used as template. In fact, the centromeric signal was more intense than the signal on the secondary constriction (see insert in Figure 3A). Secondly, when the painting probe used was XTR-7p (which did not include the secondary constriction or rDNA sequences), the hybridization signal observed on XLA3L was similar to that obtained when XTR-7w was used (Figure 3C,D). Since this probe did not include the secondary constriction of chromosome 7 from *X. tropicalis*, it can be assumed that it could not detect rDNA on the short arm of XLA3L. Finally, the hybridization pattern of an rDNA probe on *X. laevis* was different to the pattern obtained after chromosome painting with XTR-7w or XTR-7p probes. The hybridization signal obtained with the 18S + 28S rDNA probe was located specifically on the secondary constriction of chromosome 3L (Figure 4C,D), while the hybridization signal obtained with XTR-7w probes was located on the short arm of this chromosome, but not on the secondary constriction (Figure 4E,F).

This evidence reveals the existence of common sequences in XTR7 and the short arm of XLA3L that could be associated with a common ancestral location of the NOR. Comparative cytological analyses in species of the genus *Xenopus* revealed that functional NOR is present in only one homologous pair. The NOR-bearing chromosome is highly variable in *Xenopus* species [27], a characteristic observed in many other amphibian taxa with conserved karyotypes (reviewed by [49,63]). The wide relocation of the NOR observed in the Pipidae family has hampered the identification of the ancestral *loci* of the NOR, while the conservation of linkage groups between species indicates that simple rearrangements, such as translocations, are not involved in NOR relocation [64]. The common sequences shared between XTR7 and XLA3L may be a remnant of minute insertions involving the NOR. It is also possible that the positive signals observed after painting with XTR-7w and XTR-7p probes were due to conserved DNA sequences that were differentially amplified in the karyotypes of both species. It should be noted that the short arms of chromosomes 3L and 3S from *X. laevis* are formed by late-replicating, C-positive heterochromatin [6], and they stained intensely when *X. laevis* chromosomes hybridized to genomic or Cot DNA from *X. tropicalis*. These sequences could be different to those identified by GISH and FISH with Cot DNA, since painting probes did not reveal any signals on the short arm of XLA3S (or XLA4L). Additionally, the genomic DNA of *X. laevis* did not reveal any region of repeat DNA in the *X. tropicalis* karyotype apart from the chromosome ends. Thus, if repetitive sequences are shared between XTR7 and XLA3L, there must be a lower amount in *X. tropicalis* than in *X. laevis*. To test these hypotheses, it will be of interest to check if the XTR-7w probe also paints the NOR-bearing chromosomes in other *Xenopus* species, or if the short arm of chromosome 3L from *X. laevis* paints metaphase chromosomes from *X. tropicalis*.

Previous painting experiments in *X. laevis* with XTR7 also revealed a positive signal on the short arm of one acrocentric chromosome that was identified as chromosome 13 (4L according to [32]) [29,65]. We did not observe a positive signal on this chromosome pair in the samples we analyzed. It was proposed that the positive signal on 4L was due to rDNA sequences [29]. Our FISH experiments to detect rDNA in both *X. tropicali*s and *X. laevis* did not reveal other positive signals, apart from those located on the secondary constriction of those species (Figure 4A,B). Using different rDNA probes, other authors have observed additional positive signals in the karyotype of *X. tropicalis* (the ends of the long arms of chromosomes 6 and 9 and the ends of the short arm of chromosome 7) that have been attributed to rDNA sequences [66]. The differences between our results and those obtained by others could be explained by population differences in the location of rDNA sequences, as the NOR in *Xenopus* is highly mutable and has the capability for rapid evolutionary changes [27]. Alternatively, the signals attributed to rDNA may be due to sequences other than 18S + 28S, since the probe was generated and labeled by PCR from genomic DNA [66], or more probably due to fluorescence leakage from the 5S probe, since the signals coincide with strong signals from the 5S probe [66].

Alternatively, the intense signal observed by [29] on the short arm of one XLA4L could be due to the accumulation of repetitive sequences on this chromosome (positive regions revealed in this work by GISH and FISH with Cot DNA in *X. laevis*). Additional evidence in support of this hypothesis is that [29] only detected the signal in one chromosome, while we observed differences in the intensity of the GISH/Cot signal when the homologous XLA3S (or XLA4L) were compared. We probably did not paint this chromosome with XTR-7w, because we used high amounts of Cot-1 DNA as competitor DNA instead of genomic DNA.

As for the less intense and scattered signals observed on most *X. laevis* chromosomes, it has also been proposed that they correspond to additional copies of rDNA. While our FISH experiments rule out that these signals correspond to 18S + 28S rDNA sequences, they could be due to 5s rDNA, which is distributed at the ends of the long arms of almost all *X. laevis* chromosomes [9]. However, if this was the case, the XTR7 painting probe should also detect the 5S rDNA in *X. tropicalis*.

## 4. Conclusions

Subtelomeric regions in *X. tropicalis* and *X. laevis* chromosomes were highly enriched in common repetitive sequences. Other blocks of repetitive DNA in *X. tropicalis* were located on the short arms of chromosomes 3 and 8, two regions with a scarcity of genetic markers that also show assembly problems; and on the C-positive regions corresponding to the short arm of chromosome 4 and the secondary constriction of chromosome 9. The repetitive sequences located on the short arm of chromosome 3 from *X. tropicalis* were not related to the sequences on the short arm of chromosomes 3L and 3S from *X. laevis*, although these chromosomes are homoeologous, indicating that these regions evolved independently in these species. Furthermore, all the other repetitive sequences in *X. tropicalis* and *X. laevis* may be species specific, as they were not revealed in cross-species hybridizations.

Painting experiments with chromosome 7 from *X. tropicalis* also revealed interesting relationships between *X. tropicalis* and *X. laevis* karyotypes. XTR-7w and XTR-7p probes painted the homologous chromosomes in *X. laevis* (7L and 7S), together with an intense signal on the short arm of chromosome 3L. Although the NOR was located on this region, the painting signal did not correspond to the location of 18S + 28S rDNA sequences, but to a region that shares sequence homology with *X. tropicalis* chromosome 7 (the signal was also visible using the XTR-7p probe). These results also confirm that the repeated sequences on the short arm of XTR3 were not related to those located on XLA3S, since no positive signal was identified on XTR3 after painting with XTR-7p and XTR-7w probes.

**Figure 5:**
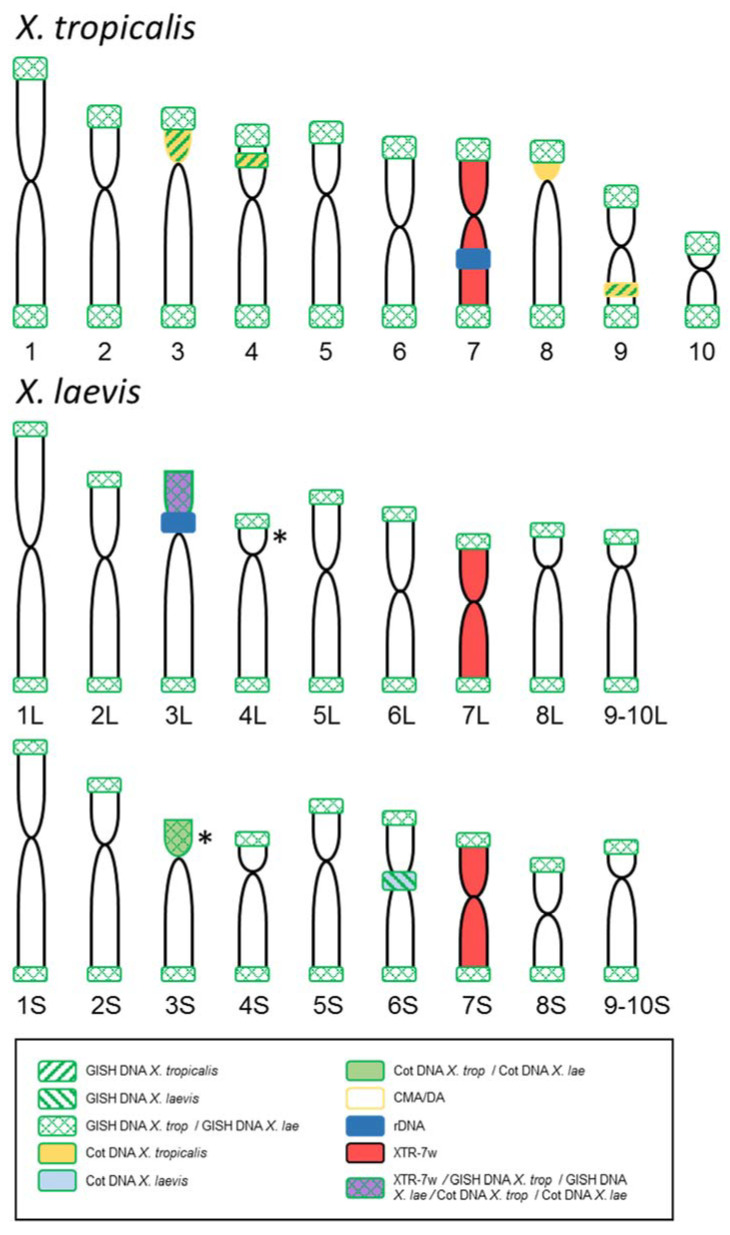
Summary of the results obtained by GISH, FISH and chromosome painting comparing the signals observed in the karyotypes of *X. tropicalis* and *X. laevis*. The probes used and the color codes are indicated in the box. (*) Alternative possibilities for a positive signal identified by GISH/Cot with chromosomes and probes from *X. laevis* (represented only in XLA3S; according to chromosome morphology could be on XLA4L instead). The size and shape of the chromosomes of both species are based on the ideograms by [28], but the nomenclature follows [9] for *X. tropicalis* and [32] for *X. laevis*.

The conclusions of the cytogenetic analysis carried out on *X. tropicalis* and *X. laevis* karyotypes are summarized in Table 1 and Figure 5.

## Figures and Tables

**Table 1:** Summary of the results obtained by different cytogenetic techniques on the chromosomes of *X. tropicalis* and *X. laevis*.

XTR [9]	XTR [62]	XTR [28]	XTR [25]	Morph.	GISH	Cot	CMA/DA	XLA [32]	XLA [26]	GISH/Cot	XTR-7w
XTR1	1	1	1	S	T	T		XLA1L	1	T	
								XLA1S	2	T	
XTR2	2	2	2	S	T	T		XLA2L	3	T	
								XLA2S	8	T	
XTR3	3	9	9	A	T, p	T, p	p	XLA3L (N)	12	T, p	p
								XLA3S	16	T, p (pol) ^1^	
XTR4	5	3	4	S	T, c2 p	T, c2 p		XLA4L	13	T, p (pol) ^1^	
								XLA4S	17	T	
XTR5	4	4	3	S	T	T		XLA5L	4	T	
								XLA5S	5	T	
XTR6	6	8	8	M	T	T		XLA6L	9	T	
								XLA6S	6	T, C (pol)	
XTR7 (N)	7	5	5	S	T	T	N	XLA7L	7	T	w
								XLA7S	10	T	w
XTR8	8	10	10	A	T	T, p		XLA8L	14	T	
								XLA8S	11	T	
XTR9	9	6	6	S	T, c2 q	T, c2 q	c2 q	XLA9L	15	T	
XTR10	10	7	7	S	T	T					
								XLA9S	18	T	
							

XTR [9]: Nomenclature of *X. tropicalis* chromosomes used in this study; XTR [62], XTR [28] and XTR [25]: alternative nomenclatures for *X. tropicalis* chromosomes, according to the references indicated in each column; Morph: chromosome morphology; GISH: results of GISH in *X. tropicalis* with a gDNA probe from the same species; Cot: results of FISH in *X. tropicalis* with Cot DNA from the same species; CMA/DA: signal in *X. tropicalis* after triple staining with CMA_3_/DA/DAPI; XLA [32]: nomenclature of *X. laevis* chromosomes used in this study; XLA [26]: alternative nomenclature for *X. laevis* chromosomes according to [26]; GISH/Cot: results in *X. laevis* after GISH and FISH with Cot DNA from *X. laevis*; XTR-7w: chromosome painting in *X. laevis* with the XTR-7w probe. Abbreviations: p: short arm; q: long arm; c2: secondary constriction; C: centromere; N: NOR; T: telomere; pol: polymorphic; w: whole chromosome. Equivalence between *X. tropicalis* and *X. laevis* chromosomes was established according to [32]. Chromosome morphology: Metacentric (M) (1 > q/p > 1.17); Submetacentric (S) (1.2 > q/p > 2.8); Acrocentric (A) (2.3 > q/p > 5.7); Telocentric (T). ^1^ Alternative possibilities for the positive signal identified by GISH with chromosomes and probe from *X. laevis*: the proposed signal in XLA3S could be in XLA4L instead.

## Data Availability

The data presented in this study are available in the supplementary materials (Figures S1–S5).

## Supplementary Materials

The following are available online at https://www.mdpi.com/article/10.3390/genes12050617/s1—Figure S1: In situ hybridization on metaphase spreads from *X. tropicalis* using genomic DNA labeled with biotin 11–dUTP as probe; Figure S2: Comparisons of male and female chromosomes from *X. tropicalis* after GISH with male and female probes from the same species; Figure S3: In situ hybridization on metaphase spreads from *X. tropicalis* using Cot-1 to Cot-16 DNA from *X. tropicalis*; Figure S4: Comparison of GISH results on different *X. laevis* individuals; Figure S5: Complete metaphase plates showing the chromosomes included in Figure 4C–F.

## References

[1] Zlotina A., Dedukh D., Krasikova A. (2017). Amphibian and avian karyotype evolution: Insights from lampbrush chromosome studies. Genes.

[2] Olmo E. (1991). Genome variations in the transition from amphibians to reptiles. J. Mol. Evol..

[3] Canapa A., Barucca M., Biscotti M.A., Forconi M., Olmo E. (2016). Transposons, genome size, and evolutionary insights in animals. Cytogenet. Genome Res..

[4] Schmid M., Evans B.J., Bogart J.P. (2015). Polyploidy in Amphibia. Cytogenet. Genome Res..

[5] Rodriguez F., Arkhipova I.R. (2018). Transposable elements and polyploid evolution in animals. Curr. Opin. Genet. Dev..

[6] Schmid M., Steinlein C. (1991). Chromosome banding in Amphibia XVI. High-resolution replication banding patterns in *Xenopus laevis*. Chromosoma.

[7] Schmid M. (1978). Chromosome Banding in Amphibia. II. Constitutive Heterochromatin and Nucleolus Organizer Regions in Ranidae, Microhylidae and Rhacophoridae. Chromosoma.

[8] Schempp W., Schmid M. (1981). Chromosome Banding in Amphibia VI. BrdU-Replication Patterns in Anura and Demonstration of XX/XY Sex Chromosomes in *Rana esculenta*. Chromosoma.

[9] Schmid M., Steinlein C. (2015). Chromosome Banding in Amphibia. XXXII. The Genus *Xenopus* (Anura, Pipidae). Cytogenet. Genome Res..

[10] Ashman T.-L., Bachtrog D., Blackmon H., Goldberg E.E., Hahn M.W., Kirkpatrick M., Kitano J., Mank J.E., Mayrose I., Ming R. (2014). Tree of sex: A database of sexual systems. Sci. Data.

[11] Schmid M., Nanda I., Steinlein C., Kausch K., Haaf T., Epplen J.T. (1991). Sex-Determining Mechanisms and Sex Chromosomes in Amphibia. Amphibian Cytogenetics and Evolution.

[12] Zattera M.L., Gazolla C.B., de Soares A., Gazoni T., Pollet N., Recco-Pimentel S.M., Bruschi D.P. (2020). Evolutionary Dynamics of the Repetitive DNA in the Karyotypes of *Pipa carvalhoi* and *Xenopus tropicalis* (Anura, Pipidae). Front. Genet..

[13] de Paula Bueno G., Gatto K.P., Gazolla C.B., Leivas P.T., Struett M.M., Moura M., Bruschi D.P. (2021). Cytogenetic characterization and mapping of the repetitive DNAs in *Cycloramphus bolitoglossus* (Werner, 1897): More clues for the chromosome evolution in the genus *Cycloramphus* (Anura, Cycloramphidae). PLoS ONE.

[14] Charlesworth D., Charlesworth B., Marais G.A.B. (2005). Steps in the evolution of heteromorphic sex chromosomes. Heredity.

[15] Devi J., Ko J.M., Seo B.B. (2005). FISH and GISH: Modern cytogenetic techniques. Indian J. Biotechnol..

[16] Kato A., Vega J.M., Han F., Lamb J.C., Birchler J.A. (2005). Advances in plant chromosome identification and cytogenetic techniques. Curr. Opin. Plant Biol..

[17] Barzotti R., Pelliccia F., Rocchi A. (2000). Sex chromosome differentiation revealed by genomic in-situ hybridization. Chromosome Res..

[18] Yoshido A., Marec F., Sahara K. (2005). Resolution of sex chromosome constitution by genomic in situ hybridization and fluorescence in situ hybridization with (TTAGG)n telomeric probe in some species of Lepidoptera. Chromosoma.

[19] Traut W. (1999). The evolution of sex chromosomes in insects: Differentiaion of sex chromosomes in flies and moths. Eur. J. Entomol..

[20] Roco Á.S., Olmstead A.W., Degitz S.J., Amano T., Zimmerman L.B., Bullejos M. (2015). Coexistence of Y, W, and Z sex chromosomes in *Xenopus tropicalis*. Proc. Natl. Acad. Sci. USA.

[21] Furman B.L.S., Cauret C.M.S., Knytl M., Song X.Y., Premachandra T., Ofori-Boateng C., Jordan D.C., Horb M.E., Evans B.J. (2020). A frog with three sex chromosomes that co-mingle together in nature: *Xenopus tropicalis* has a degenerate W and a Y that evolved from a Z chromosome. PLoS Genet..

[22] Furman B.L.S., Evans B.J. (2016). Sequential Turnovers of Sex Chromosomes in African Clawed Frogs (*Xenopus*) Suggest Some Genomic Regions are Good at Sex Determination. G3 Genes Genomes Genet..

[23] Furman B.L.S., Evans B.J. (2018). Divergent Evolutionary Trajectories of Two Young, Homomorphic, and Closely Related Sex Chromosome Systems. Genome Biol. Evol..

[24] Evans B.J. (2008). Genome evolution and speciation genetics of clawed frog (Xenopus and Silurana). Front. Biosci..

[25] Tymowska J. (1973). Karyotype analysis of *Xenopus tropicalis* Gray, Pipidae. Cytogenet. Cell Genet..

[26] Tymowska J., Kobel H.R. (1972). Karyotype analysis of *Xenopus muelleri* (Peters) and *Xenopus laevis* (Daudin), Pipidae. Cytogenet. Genome Res..

[27] Tymowska J. (1991). Polyploidy and Cytogenetic Variation in Frogs of the Genus Xenopus. Amphibian Cytogenetics and Evolution.

[28] Uno Y., Nishida C., Yoshimoto S., Ito M., Oshima Y., Yokoyama S., Nakamura M., Matsuda Y. (2008). Diversity in the origins of sex chromosomes in anurans inferred from comparative mapping of sexual differentiation genes for three species of the Raninae and Xenopodinae. Chromosome Res..

[29] Krylov V., Kubickova S., Rubes J., Macha J., Tlapakova T., Seifertova E., Sebkova N. (2010). Preparation of *Xenopus tropicalis* whole chromosome painting probes using laser microdissection and reconstruction of *X. laevis* tetraploid karyotype by Zoo-FISH. Chromosome Res..

[30] Khokha M.K., Krylov V., Reilly M.J., Gall J.G., Bhattacharya D., Cheung C.Y.J., Kaufman S., Lam D.K., Macha J., Ngo C. (2009). Rapid gynogenetic mapping of Xenopus tropicalis mutations to chromosomes. Dev. Dyn..

[31] Wells D.E., Gutierrez L., Xu Z., Krylov V., Macha J., Blankenburg K.P., Hitchens M., Bellot L.J., Spivey M., Stemple D.L. (2011). A genetic map of *Xenopus tropicalis*. Dev. Biol..

[32] Matsuda Y., Uno Y., Kondo M., Gilchrist M.J., Zorn A.M., Rokhsar D.S., Schmid M., Taira M. (2015). A New Nomenclature of *Xenopus laevis* Chromosomes Based on the Phylogenetic Relationship to *Silurana/Xenopus* tropicalis. Cytogenet. Genome Res..

[33] Session A.M., Uno Y., Kwon T., Chapman J.A., Toyoda A., Takahashi S., Fukui A., Hikosaka A., Suzuki A., Kondo M. (2016). Genome evolution in the allotetraploid frog Xenopus laevis. Nature.

[34] Mikamo K., Witschi E. (1966). The mitotic chromosomes in *Xenopus laevis* (Daudin): Normal, sex reversed and female WW. Cytogenetics.

[35] Uno Y., Nishida C., Takagi C., Ueno N., Matsuda Y. (2013). Homoeologous chromosomes of *Xenopus laevis* are highly conserved after whole-genome duplication. Heredity.

[36] Olmstead A.W., Lindberg-Livingston A., Degitz S.J. (2010). Genotyping sex in the amphibian, *Xenopus* (*Silurana*) *tropicalis*, for endocrine disruptor bioassays. Aquat. Toxicol..

[37] Bewick A.J., Chain F.J.J., Zimmerman L.B., Sesay A., Gilchrist M.J., Owens N.D.L., Seifertova E., Krylov V., Macha J., Tlapakova T. (2013). A Large Pseudoautosomal Region on the Sex Chromosomes of the Frog *Silurana tropicalis*. Genome Biol. Evol..

[38] Yoshimoto S., Okada E., Umemoto H., Tamura K., Uno Y., Nishida-Umehara C., Matsuda Y., Takamatsu N., Shiba T., Ito M. (2008). A W-linked DM-domain gene, DM-W, participates in primary ovary development in *Xenopus laevis*. Proc. Natl. Acad. Sci. USA.

[39] Tymowska J., Fischberg M. (1982). A comparison of the karyotype, constitutive heterochromatin, and nucleolar organizer regions of the new tetraploid species *Xenopus epitropicalis* Fischberg and Picard with those of *Xenopus tropicalis* Gray (Anura, Pipidae). Cytogenet. Cell Genet..

[40] Schmid M., Haaf T., Geile B., Sims S. (1983). Chromosome banding in Amphibia VIII. An unusual XY[XX-sex chromosome system in *Gastrotheca riobambae* (Anura, Hylidae). Chromosoma.

[41] Green D.M. (1988). Cytogenetics of the endemic New Zealand frog, *Leiopelma hochstetteri*: Extraordinary supernumerary chromosome variation and a unique sex-chromosome system. Chromosoma.

[42] Schmid M., Steinlein C. (2003). Chromosome banding in Amphibia: XXIX. The primitive XY/XX sex chromosomes of *Hyla femoralis* (Anura, Hylidae). Cytogenet. Genome Res..

[43] Schmid M., Ohta S., Steinlein C., Guttenbach M. (1993). Chromosome banding in Amphibia XIX. Primitive ZW/ZZ sex chromosomes in *Buergeria buergeri* (Anura, Rhacophoridae). Cytogenet. Cell Genet..

[44] Abramyan J., Ezaz T., Marshall Graves J.A., Koopman P.A. (2009). Z and W sex chromosomes in the cane toad (*Bufo marinus*). Chromosome Res..

[45] Sinzelle L., Thuret R., Hwang H.-Y., Herszberg B., Paillard E., Bronchain O.J., Stemple D.L., Dhorne-Pollet S., Pollet N. (2012). Characterization of a novel *Xenopus tropicalis* cell line as a model for in vitro studies. Genesis.

[46] Nieuwkoop P.D., Faber J. (1994). Normal Table of Xenopus laevis (Daudin): A Systematical and Chronological Survey of the Development from the Fertilized Egg Till the End of Metamorphosis.

[47] Krylov V., Tlapakova T., Macha J. (2007). Localization of the single copy gene *Mdh2* on Xenopus tropicalis chromosomes by FISH-TSA. Cytogenet. Genome Res..

[48] Schnedl W., Abraham R., Dann O., Geber G., Schweizer D. (1981). Preferential fluorescent staining of heterochromatic regions in human chromosomes 9, 15, and the Y by D 287/170. Hum. Genet..

[49] Schmid M., Steinlein C., Bogart J.P., Feichtinger W., León P., La Marca E., Díaz L.M., Sanz A., Chen S.H., Hedges S.B. (2010). The chromosomes of terraranan frogs: Insights into vertebrate cytogenetics. Cytogenet. Genome Res..

[50] Al-Rikabi A., Liehr L.B., Liehr T. (2020). Glass needle-based chromosome microdissection—How to set up probes for molecular cytogenetics?. Video J. Clin. Res..

[51] Trifonov V., Vorobieva N.N., Rens W., Liehr T. (2009). FISH with and Without COT1 DNA. Fluorescence In Situ Hybridization (FISH)—Application Guide.

[52] Telenius H., Carter N.P., Bebb C.E., Nordenskjöld M., Ponder B.A.J., Tunnacliffe A. (1992). Degenerate oligonucleotide-primed PCR: General amplification of target DNA by a single degenerate primer. Genomics.

[53] Endow S.A. (1982). Polytenization of the ribosomal genes on the X and Y chromosomes of *Drosophila melanogaster*. Genetics.

[54] Schweizer D. (1976). Reverse fluorescent chromosome banding with chromomycin and DAPI. Chromosoma.

[55] Tymowska J., Fischberg M. (1973). Chromosome complements of the genus *Xenopus*. Chromosoma.

[56] Suda M., Uno Y., Mori Y., Matsuda Y., Nakamura M. (2011). Molecular cytogenetic characterization of telomere-specific repetitive DNA sequences in *Rana rugosa*. J. Exp. Zool. A Ecol. Genet. Physiol..

[57] Spohr G., Reith W., Sures I. (1981). Organization and sequence analysis of a cluster of repetitive DNA elements from *Xenopus laevis*. J. Mol. Biol..

[58] Cioffi M.B., Kejnovsky E., Bertollo L.A.C. (2011). The chromosomal distribution of microsatellite repeats in the genome of the wolf fish *Hoplias malabaricus*, focusing on the sex chromosomes. Cytogenet. Genome Res..

[59] De Oliveira T.D., Kretschmer R., Bertocchi N.A., Degrandi T.M., De Oliveira E.H.C., De Cioffi M.B., Garnero A.D.V., Gunski R.J. (2017). Genomic organization of repetitive DNA in woodpeckers (Aves, Piciformes): Implications for karyotype and ZW sex chromosome differentiation. PLoS ONE.

[60] Tashiro S., Nishihara Y., Kugou K., Ohta K., Kanoh J. (2017). Subtelomeres constitute a safeguard for gene expression and chromosome homeostasis. Nucleic Acids Res..

[61] Kwapisz M., Morillon A. (2020). Subtelomeric Transcription and its Regulation. J. Mol. Biol..

[62] Mitros T., Lyons J.B., Session A.M., Jenkins J., Shu S., Kwon T., Lane M., Ng C., Grammer T.C., Khokha M.K. (2019). A chromosome-scale genome assembly and dense genetic map for *Xenopus tropicalis*. Dev. Biol..

[63] Schmid M., Steinlein C., Bogart J.P., Feichtinger W., Haaf T., Nanda I., Del Pino E.M., Duellman W.E., Hedges S.B. (2012). The hemiphractid frogs: Phylogeny, embryology, life history, and cytogenetics. Cytogenet. Genome Res..

[64] Mezzasalma M., Glaw F., Odierna G., Petraccioli A., Guarino F.M. (2015). Karyological analyses of *Pseudhymenochirus merlini* and *Hymenochirus boettgeri* provide new insights into the chromosome evolution in the anuran family Pipidae. Zool. Anz..

[65] Krylov V., Tlapakova T. (2015). *Xenopus* Cytogenetics and Chromosomal Evolution. Cytogenet. Genome Res..

[66] Knytl M., Smolík O., Kubíčková S., Tlapáková T., Evans B.J., Krylov V. (2017). Chromosome divergence during evolution of the tetraploid clawed frogs, *Xenopus mellotropicalis* and *Xenopus epitropicalis* as revealed by Zoo-FISH. PLoS ONE.

