# Supplementary material for: Comparative Distribution of Repetitive Sequences in the Karyotypes of Xenopus tropicalis and Xenopus laevis (Anura, Pipidae)
Source: Genes (Basel). 2021 Apr 21;12(5):617. doi: 10.3390/genes12050617 (PMC8143290; doi:10.3390/genes12050617)
Supplement: Supplementary file 1 [file genes-12-00617-s001.zip › genes-1184874-supplementary.pdf]

Figure S1

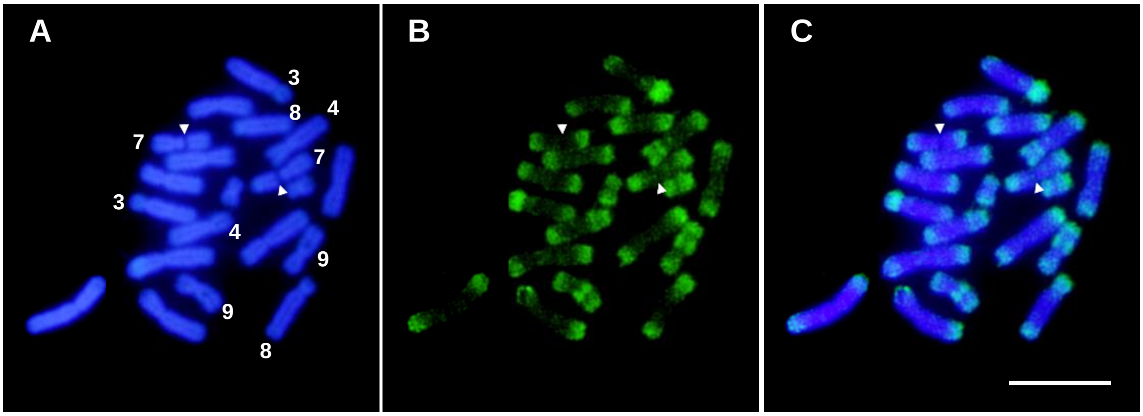

Figure S1. In situ hybridization on metaphase spreads from a female *X. tropicalis* using genomic DNA labeled with biotin 11–dUTP as probe: A) DAPI staining, B) FISH with labeled genomic DNA detected using three rounds of immunological amplification, C) merge. The arrowheads point to the secondary constriction on pair 7 (NOR). Scale: 5 µm.

Figure S2

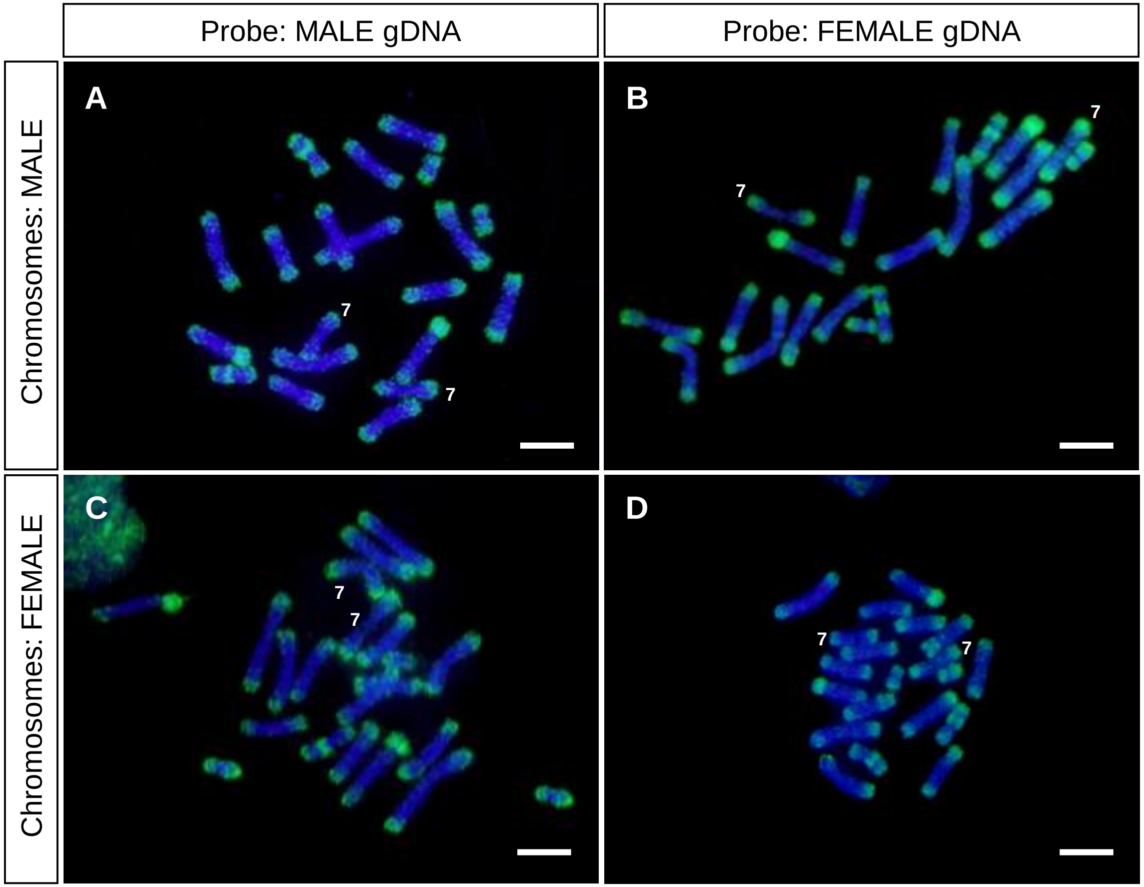

Figure S2. Metaphase chromosomes from male (A and B) female (C and D) from *X. tropicalis*, hybridized using as probe genomic DNA from male (A and C) and female (B and D). The sex chromosomes (XTR7) are indicated. No differences between male and female metaphases and/or probes were identified. Scale: 5 µm.

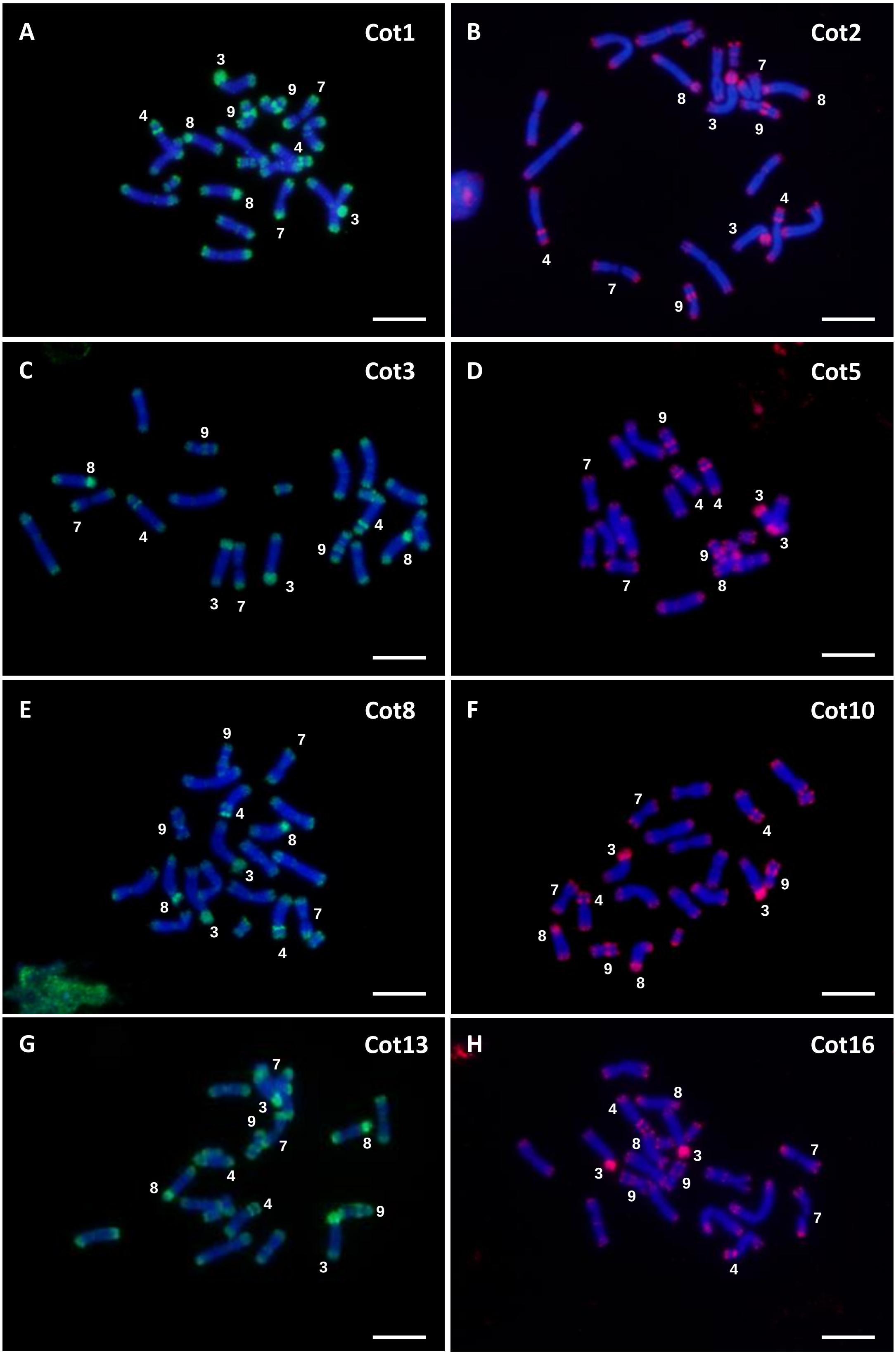

Figure S3. In situ hybridization on metaphase spreads from *X. tropicalis* (female) using Cot DNA (female) labeled with Texas Red–dUTP (B, D, F and H) or SpectrumGreen–dUTP (A, C, E and G) as probes. A) DNA Cot1; B) DNA Cot2; C) DNA Cot3; D) DNA Cot5; E) DNA Cot8; F) DNA Cot 10; G) DNA Cot13 and H) DNA Cot16. Direct fluorescence is observed in all cases. One XTR8 is missing from figure D. Scale: 5 µm.

Figure S4

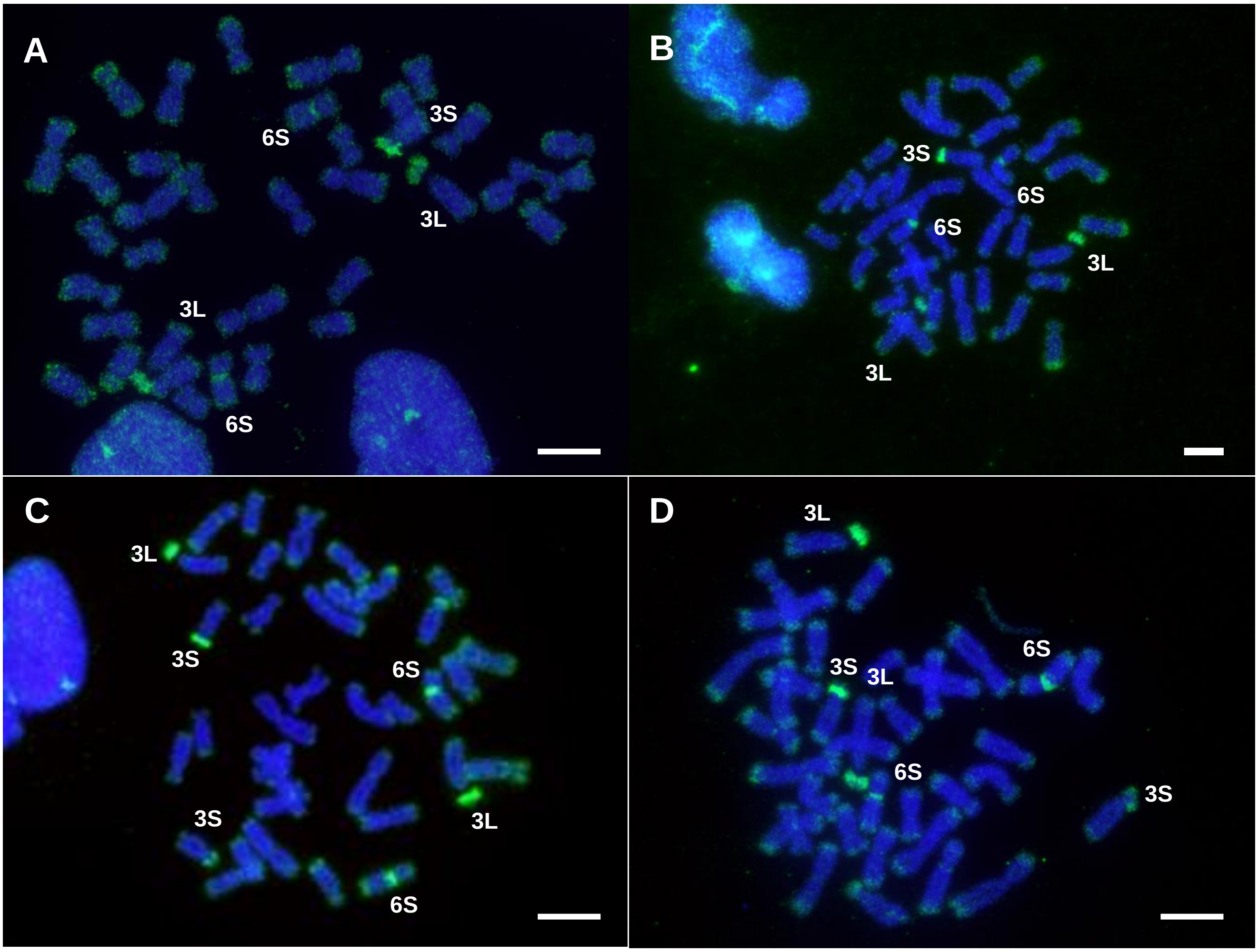

Figure S4. A-C) GISH on female chromosomes from *X. laevis* using male gDNA from *X. laevis*. D) FISH on female chromosomes from *X. laevis* using male Cot DNA from *X. laevis*. Differences between three individuals (A, B and C) in the intensity of the signal on the short arm of chromosome XLA3S and on the centromere of XLA6S. Images in C and D are from the same individual. Scale: 5 µm.

Figure S5

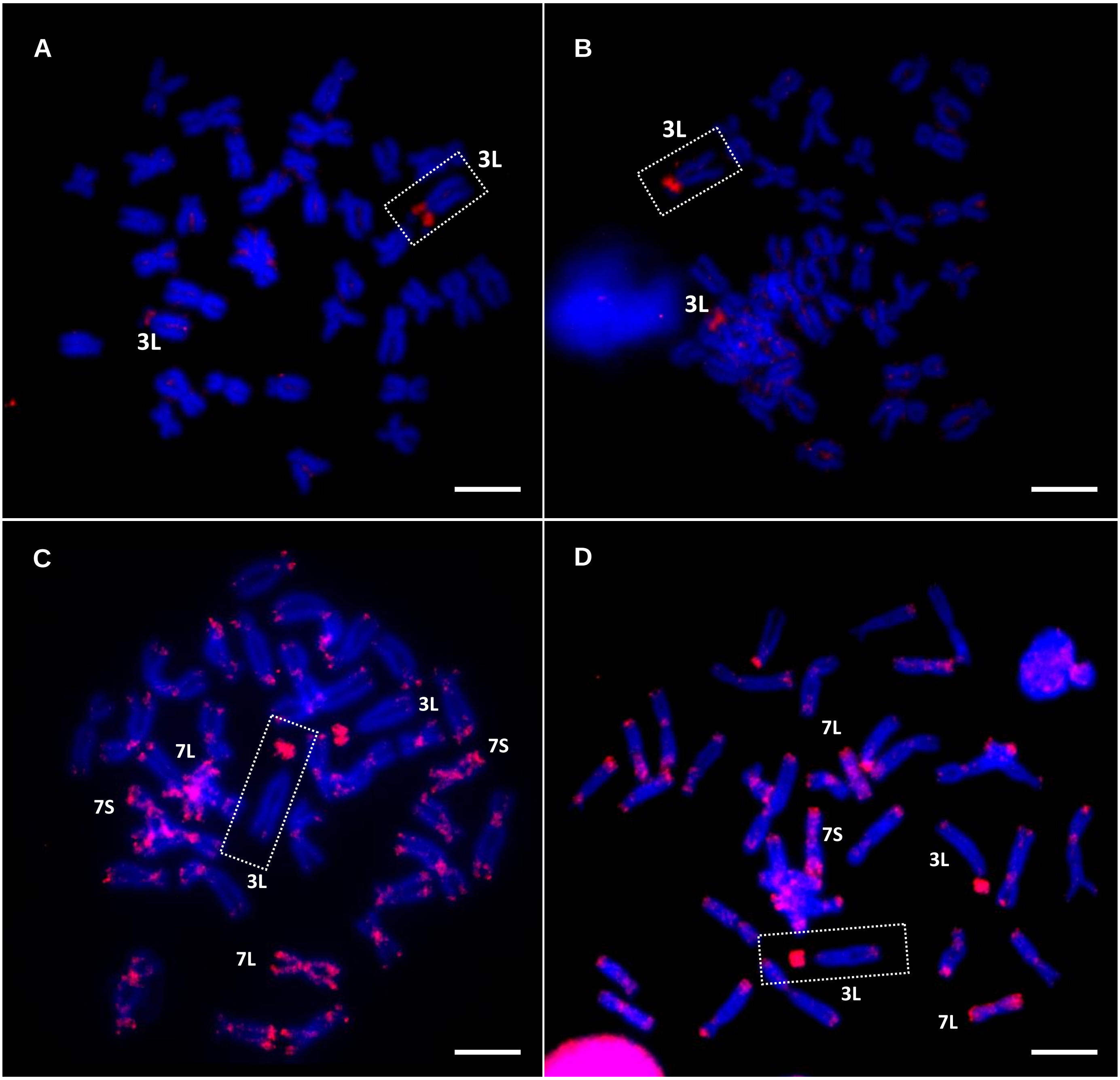

Figure S5. Metaphase plates from *X. laevis* male (A and B) and female samples (C and D) after FISH hybridization with a rDNA probe (A and B) or chromosome painting with XTR-7w (C and D). The inserts in A, B C and D correspond to the chromosomes in figure 4C, 4D, 4E and 4F respectively. Scale: 5 µm.
